# Evaluating the feasibility and efficacy of exercise interventions for older survivors of cancer

**DOI:** 10.1002/cncr.70278

**Published:** 2026-03-01

**Authors:** Jingran Ji, Arman Niknafs, Lee W. Jones

**Affiliations:** 1 Department of Medicine, Hematology/Oncology, David Geffen School of Medicine at the University of California, Los Angeles, California, USA; 2 Division of Outcomes Research, Department of Population Sciences, Beckman Research Institute, City of Hope, Duarte, California, USA

Exercise interventions have consistently demonstrated benefit among survivors of cancer and are strongly recommended by multiple clinical guidelines as part of survivorship care.^1–3^ Among older survivors of cancer, studies have further shown that exercise improves physical fitness, psychological health, and quality of life.^4–6^ However, these studies in older adults are mostly limited to survivors of breast and prostate cancer and do not select for older survivors with functional deficits at baseline. Given that older survivors are a heterogeneous population with varying physical fitness after cancer treatment, studies focusing on those with reduced exercise capacity are necessary to better understand the unique challenges of this patient population.

In the article that accompanies this editorial, Brown and colleagues^7^ addressed these knowledge gaps in a secondary analysis of the multicenter Lifestyle Interventions and Independence for Elders (LIFE) study. The original LIFE study prospectively randomized sedentary and functionally limited older adults 70–89 years old to an exercise intervention versus health education alone.^8^ Sedentary was defined as having less than 125 minutes per week of moderate-intensity physical activity, and functional limitation was defined as a short physical performance battery (SPPB) score of ≤9. The secondary analysis conducted by Brown et al.^7^ focused on a subgroup of participants with a self-reported history of cancer, a total of 371 participants. The end points of interest were the change in the SPPB and the 400-meter walk speed from baseline to 6, 12, and 24 months. The exercise intervention targeted 150 minutes of moderate intensity walking (as measured by the Borg RPE) weekly, with 10 minutes of lower extremity strength training and 10 minutes of balance training at each session for a median duration of 2.6 years (interquartile range, 2.3–3.1 years). Participants attended two supervised center-based sessions per week and performed home-based exercises three to four times weekly throughout the duration of the study. Participants were allowed to start below the target exercise duration and escalate in intensity over the first 2–3 weeks. The control health education program consisted of weekly workshops for 26 weeks followed by monthly sessions that covered topics tailored to older adults (such as transportation safety, age-specific preventive care recommendations, financial health for older adults, etc.) but did not focus on increasing physical activity.

The analysis showed that the exercise intervention led to consistent improvements in the continuous SPPB score over the health education group at all three time points (0.45 points at 6 months [95% CI, 0.05–0.86, *p* = .027], 0.43 points at 12 months [95% CI, 0.02–0.83, *p* = .039], and 0.45 points at 24 months [95% CI, 0.04–0.87, *p* = .031]). The absolute increase in SPPB score from baseline in the intervention arm was 1.14 (standard error [SE] = 0.15) at 6 months, 0.91 (SE = 0.15) at 12 months, and 0.63 (SE = 0.15) at 24 months. A categorical increase in SPPB (i.e., from moderate to good performance) was only seen at 6 months and not at 12 or 24 months. The exercise intervention also led to more improvement over health education in the 400-meter walk speed at 6 months (0.042 m/s; 95% CI, 0.017–0.066, *p* < .001) and 12 months (0.036 m/s; 95% CI, 0.011–0.060, *p* = .004). However, at 24 months (0.013 m/s; 95% CI, –0.012 to 0.038, *p* = .3), there was no longer a difference between groups.

The findings of Brown et al.^7^ add to the existing literature by supporting the benefits of a supervised exercise program for older survivors of cancer, specifically focusing on those who are sedentary with physical function limitations at baseline. This study had several strengths. The selected end points are well validated measures of physical function in older adults, and the findings provide objective evidence of physical function improvement in the exercise arm over the control arm. These measures were also collected across multiple time points spanning up to 2 years, which provided important insights on the sustainability of the physical function improvements. The results demonstrated that most of the benefits in physical function improvement were in the first 6 months with a loss of efficacy over time. This was especially true for the 400-meter walk speed, where the change in the intervention group and the control group equalized at 24 months. Finally, the study had a relatively large sample size with a diverse range of cancer types including colorectal, uterine/endometrial, and lung cancer, which may improve the generalizability of these findings.

However, several limitations also need to be considered when interpreting these data. First, this was an unplanned analysis of the parent trial. Second, although statistically significant, the observed effect sizes associated with the exercise intervention were small, only marginally meeting the definition of a minimum clinically meaningful change,^9, 10^ and not sustained at 24 months. The small effect size could, in part, be due to the nature of the exercise intervention. That is, the intervention encouraged subjects to reach 150 minutes of physical activity but did not test a fully structured prescription of exercise at a specific dose (frequency × duration × intensity) and only had partial supervision to verify compliance. Relatedly, eligible participants were only required to report less than 125 minutes per week of moderate physical activity to be considered sedentary. Hence, for those participants whose baseline physical activity was already close to or at 125 minutes per week, the physical activity goal of 150 minutes was only a small increase from baseline and likely not sufficient to confer greater benefit. It was also not clear how quickly participants were able to progress to the 150-minute goal, and using the Borg scale alone to measure exercise intensity may not have led to a uniform exercise intensity over time. Finally, even though adherence was reported at 71%, the definition of adherence only captured the attendance of the in-person sessions and not the homebased activities that participants were expected to perform three to four times per week.

Notwithstanding these limitations, Brown and colleagues^7^ are one of the first to report the impact of an exercise intervention among a cohort of sedentary older survivors of cancer who have objectively measured physical function impairment. This study showed that an exercise intervention over a median duration of 2.6 years is not only safe and feasible but also led to at a least short-term benefit in physical function when measured by SPPB and 400-meter walk speed. As stated by the authors, the findings provide preliminary evidence for future prospective confirmatory trials testing the efficacy of an exercise program in frail older survivors of cancer.

To increase the clinical impact, the design of such confirmatory trials will need to consider several important design elements. First, studies should use stricter eligibility criteria to reduce heterogeneity of cancer type, stage, and treatment, ideally focusing on patients with previously understudied cancer types such as gastrointestinal and thoracic malignancies. Minimizing heterogeneity is important as modern cancer treatments differ significantly depending on the type of cancer. This results in a range of treatment toxicities and physical function impairment, which need to be accounted for in the study design and pre-planned analysis. Second, there should be greater precision regarding the timing of the exercise intervention relative to anticancer therapy initiation and/or completion. Acute and chronic toxicities of therapy will likely modify both the tolerability and the response to exercise.^11^ Third, smaller trials are required to determine the optimal type, intensity, and duration of physical activity or structured exercise to maximize both the magnitude and durability of treatment benefit. ^12–14^ Although it is not practical or feasible to conduct such a study for patients with every cancer type and treatment modality, building a strong evidence base in the most common cancers can inform larger confirmatory studies. Fourth, cancer-relevant outcomes such as disease recurrence and cancer-specific mortality should be included as secondary end points, although this may be less important in older-adult specific studies where participants may have differing priorities compared to younger populations.^15^ Fifth, correlative end points should investigate the underlying biological mechanisms driving the benefits of exercise after cancer treatment, which are not well understood.^16, 17^ These insights may guide the development of predictive biomarkers and novel mechanism-based interventions to potentiate the effects of exercise. Finally, effective implementation strategies are needed to improve access to supervised exercise programs, especially for older adults who have additional barriers such as difficulties with transportation, limited social support, and increased risk of injury.

Indeed, several ongoing trials are prospectively evaluating exercise interventions in older adults with cancer. The BREACE trial (NCT03656731)^18^ is comparing a progressive and individually tailored 12-week exercise-based intervention to usual care among older women with breast cancer receiving systemic therapy for both early and advanced-stage disease. The PACE-Mobil-PBL trial (NCT03411200)^19^ is evaluating at a 12-week multimodal intervention, including supervised exercise training, in older patients with advanced pancreatic, biliary tract, or lung cancers on first-line palliative therapy.^19^ The PROFFi trial (NCT06113016)^20^ is evaluating the potential synergistic effect of a remotely supervised exercise program in combination with a senolytic agent to reverse the accelerated-aging effects of chemotherapy in postmenopausal women treated for early-stage breast cancer. These trials will provide key data on how exercise interventions work for older adults with cancer.

In summary, this analysis reported by Brown et al.^7^ adds to an emerging body of evidence showing that the exercise interventions can improve health outcomes in older adults with cancer. Data from ongoing and future trials may further refine the specific dose of the exercise prescriptions tailored to the individual patient, identify the optimal setting for implementing exercise interventions, and ultimately improve outcomes in a large and rapidly growing population of older cancer survivors.
